# Epstein-Barr Virus Nuclear Antigen 3C Inhibits Expression of *COBLL1* and the *ADAM28-ADAMDEC1* Locus via Interaction with the Histone Lysine Demethylase KDM2B

**DOI:** 10.1128/JVI.01362-18

**Published:** 2018-10-12

**Authors:** Adam C. T. Gillman, Gillian Parker, Martin J. Allday, Quentin Bazot

**Affiliations:** a Molecular Virology, Department of Medicine, Imperial College London, London, United Kingdom; University of California, Irvine

**Keywords:** EBNA3, Epstein-Barr virus, KDM2B, histone modifications, transcriptional regulation, virology

## Abstract

Epstein-Barr virus nuclear antigen 3C (EBNA3C) is a well-defined repressor of host gene expression in B cells transformed by Epstein-Barr virus (EBV) that cooperates with various cellular factors. It is established that EBNA3C interacts with the cellular factor RBPJ (RBP-Jκ or CBF1) through two distinct motifs: the TFGC motif, also called the homology domain (HD) motif, and the VWTP motif. In this study, we investigated the role of each motif in EBNA3C transcriptional repression activity by using two novel recombinant viruses with single RBPJ interaction motifs mutated (EBNA3C HDmut and EBNA3C W227S). Infection of primary B cells with either of these recombinant EBVs led to the successful establishment of lymphoblastoid cell lines (LCLs). Gene expression analysis showed that full repression of EBNA3C target genes is not achieved by EBNA3C HDmut compared to that with EBNA3C W227S or the EBNA3C wild type (WT). Focusing on the well-characterized EBNA3C-repressed genes *COBLL1*, *ADAM28*, and *ADAMDEC1*, we investigated the mechanism of EBNA3C-mediated transcriptional repression. Chromatin immunoprecipitation (ChIP) analysis indicated that EBNA3C HDmut is still able to recruit Polycomb proteins BMI1 and SUZ12 to *COBLL1* as efficiently as EBNA3C WT does, leading to the full deposition of the repressive histone mark H3K27me3. However, we found that the activation-associated chromatin mark H3K4me3 is highly enriched at EBNA3C target genes in LCLs expressing EBNA3C HDmut. We show here that EBNA3C interacts with the histone lysine demethylase KDM2B and that this interaction is important for H3K4me3 removal and for the EBNA3C-mediated repression of *COBLL1* and the *ADAM28-ADAMDEC1* locus.

**IMPORTANCE** EBV is a virus associated with human cancers and is well known for its ability to transform B lymphocytes into continuously proliferating lymphoblastoid cell lines. EBNA3C is considered an oncoprotein and has been shown to be essential for B cell transformation by EBV. EBNA3C is well characterized as a viral transcription factor, but very little is known about its mechanisms of action. In the present study, we demonstrate that removal of the activating histone mark H3K4me3 and deposition of the repressive mark H3K27me3 by EBNA3C on *COBLL1* are achieved by at least two distinct mechanisms. Furthermore, we discovered that EBNA3C interacts with the lysine demethylase KDM2B and that this interaction is important for its transcriptional repressive function. The findings in this study provide new insights into the mechanism used by the oncoprotein EBNA3C to repress cellular target genes.

## Abstract (precis)

EBV is a virus associated with human cancers and is well known for its ability to transform B lymphocytes into continuously proliferating lymphoblastoid cell lines. EBNA3C is considered an oncoprotein and has been shown to be essential for B cell transformation by EBV. EBNA3C is well characterized as a viral transcription factor, but very little is known about its mechanisms of action. In the present study, we demonstrate that removal of the activating histone mark H3K4me3 and deposition of the repressive mark H3K27me3 by EBNA3C on *COBLL1* are achieved by at least two distinct mechanisms. Furthermore, we discovered that EBNA3C interacts with the lysine demethylase KDM2B and that this interaction is important for its transcriptional repressive function. The findings in this study provide new insights into the mechanism used by the oncoprotein EBNA3C to repress cellular target genes.

## INTRODUCTION

Epstein-Barr virus (EBV) is a large DNA virus that belongs to the gammaherpesvirus subfamily and persistently infects >90% of the human population. Despite being a ubiquitous virus, EBV is also one of the most transforming viruses identified. It is etiologically associated with a variety of B cell malignancies in humans, including Burkitt lymphoma (BL), Hodgkin's lymphoma (HL), and diffuse large B cell lymphoma (DLBCL) (1, 2). EBV is implicated in around 1 to 1.5% of worldwide cancer incidences (3). Primary infection typically occurs during the first few years of life, is asymptomatic, and leads to lifelong EBV latency. When primary infection is delayed into adolescence or adulthood, it can result in the temporarily debilitating but relatively benign condition infectious mononucleosis. *In vivo*, infection of mature B cells by EBV initially leads to their activation and differentiation into proliferating B blasts. These activated B blasts then migrate through germinal centers, where they further differentiate, resulting in resting memory B cells that carry the EBV genome as extrachromosomal episomes, forming long-lived reservoirs of EBV infection (4, 5). *In vitro*, however, EBV has the unique capacity to infect, activate, and induce the transformation of B cells, resulting in continuously proliferating lymphoblastoid cell lines (LCLs) resembling activated B blasts. LCLs express all EBV latency-associated genes, producing six EBV nuclear antigens (EBNA1, -2, -3A, -3B, and -3C and leader protein [LP]), three latent membrane proteins (LMP1, -2A, and -2B), two small noncoding RNAs (EBER1 and -2), and microRNA transcripts from the BHRF1 and BamHI A (BART) regions, which act in concert to induce and maintain continuous proliferation (2, 6, 7).

The EBNA3A, EBNA3B, and EBNA3C genes are considered to comprise a family of nonredundant EBV genes which probably arose from gene duplication during primate gammaherpesvirus evolution. Although the EBNA3 antigens possess the same genomic structure, they share only a domain of very limited amino acid sequence homology (called the homology domain [HD]) (8, 9). EBNA3C is a viral transcription factor that is essential for B cell transformation and is absolutely required for the continuous proliferation of LCLs (reviewed in reference 10). This crucial function is due to the ability of EBNA3C to repress the cyclin-dependent kinase inhibitor gene *CDKN2A* (coding for p16^INK4A^) (11–13). Interestingly, EBNA3C does not appear to bind DNA directly but is tethered to target genes by associating with DNA sequence-binding factors (10, 14, 15), one example of which is RBPJ (also known as RBP-Jκ or CBF1) (16–20). Furthermore, we recently showed that EBNA3C is also able to recruit RBPJ to target genes (21).

Deletion mutagenesis of EBNA3C mapped N-terminal residues 180 to 231 as essential residues for the interaction with RBPJ (18, 20, 22, 23). Four core residues (_209_TFGC_212_) within the homology domain of EBNA3C were identified as being important for the interaction with RBPJ. The TFGC motif is not a known RBPJ interaction motif. However, mutation of these residues to _209_AAAA_212_ (HDmut) in EBNA3C destabilized its interaction with RBPJ as determined by coimmunoprecipitation (co-IP) (22, 23). It was further shown that HDmut failed to sustain LCL proliferation when transfected into LCLs with conditional EBNA3C after inactivation of EBNA3C (22, 23), and it failed to upregulate *TCL1*, an EBNA3C target gene (23). More recently, a study by Calderwood and colleagues showed that EBNA3C HDmut was able to interact with RBPJ as efficiently as the EBNA3C wild type (WT) does (24). That study identified a RAM (RBPJ-associated molecule)-like motif, _226_VWTP_229_, upstream of the TFGC motif. The RAM motif, ΦWΦP (Φ = a hydrophobic residue), is conserved across species and is core to Notch binding of RBPJ (25, 26). Upon cognate ligand binding of the Notch receptor, a two-step proteolytic cleavage event releases the Notch intracellular domain (NICD) (27). The NICD then translocates to the nucleus and binds RBPJ via its RAM domain and ankyrin repeats (27, 28). Docking of the conserved ΦWΦP motif into the hydrophobic pocket exposed on the beta-trefoil domain of RBPJ is critical to the binding of the RAM domain (25, 26). It has been shown that mutation of either the conserved tryptophan or proline residue of the Notch ΦWΦP motif prevents RBPJ binding (29–32). The Epstein-Barr virus latent protein EBNA2 also possesses a RAM motif (PWWP), and this motif has been shown to be essential for EBNA2 interaction with RBPJ as well as for gene transactivation (33–35).

It was demonstrated for the EBNA3C protein that mutation of both motifs (_209_AAAA_212_ and W227S) was required for an effective loss of RBPJ interaction (24). Furthermore, we recently showed that a recombinant EBV with an EBNA3C protein unable to interact with RBPJ (carrying both HDmut and W227S mutations) was able to establish stable LCLs, although they proliferated very slowly compared to LCLs established with WT EBNA3C (LCL WT). This provided compelling evidence that the EBNA3C-RBPJ interaction is critical but may not be absolutely essential for LCL growth in the context of viral infection (21). Apart from its well-characterized interaction with RBPJ, EBNA3C has also been reported to interact with the transcriptional repressors Sin3A and CtBP1 in LCLs (11, 14, 36). These interactions have been shown to play a role in EBNA3C-mediated regulation of the *CDKN2A* locus.

EBNA3C is a multifunctional protein with well-characterized transcriptional repressor functions. However, the exact mechanisms by which EBNA3C regulates gene expression are still poorly understood. It is known that EBNA3C can regulate gene expression through the modulation of chromatin looping between distal regulatory elements and gene transcription start sites (TSS) (37–39). EBNA3C extensively cooperates with EBNA3A as well as EBNA3B in the regulation of thousands of cellular genes (40, 41). Furthermore, cooperation between EBNA3A and EBNA3C is known to epigenetically downregulate *CDKN2A* and *BCL2L11*, two cell genes involved in the regulation of the cell cycle and apoptosis (11, 12, 42, 43). EBNA3A and EBNA3C together are necessary to trigger the deposition of the H3K27 trimethylation (H3K27me3) epigenetic mark on the promoters of both of these tumor suppressor genes. Using an EBNA3C conditional system (EBNA3C-HT lines) in which the EBNA3C protein is functional only in the presence of the activating ligand for the modified-estrogen receptor (4-hydroxytamoxifen [HT]), we recently explored the temporal changes in epigenetic marks on *COBLL1*, a cellular gene regulated only by EBNA3C (not by EBNA3A or EBNA3B), and on the *ADAM28-ADAMDEC1* locus, which is regulated by both EBNA3A and EBNA3C. We demonstrated that EBNA3C-mediated repression of transcription involved a two-step mechanism—a rapid loss of activation-associated histone marks that led to repression of mRNA expression and then recruitment of Polycomb group (PcG) proteins and increased repressive histone H3K27me3 marks (21).

PcG proteins form two multiprotein Polycomb repressive complexes (PRC1 and PRC2). PRC1 and PRC2 are known to catalyze lysine 119 monoubiquitination of histone H2A (H2AK119ub1) and H3K27me3, respectively (44, 45). PRC2 is a multiprotein complex mediating transcriptional repression through the histone methyltransferase activity of one of its components, EZH2. Other main components of PRC2 are SUZ12, EED, and RbAp46/48. The PRC1 complex comprises 4 core subunits: the E3 ligase proteins (RING), the chromobox proteins (CBX), the polyhomeotic proteins (PHC), and Polycomb group RING finger proteins (PCGF). Interestingly, recent studies challenged the classical sequential recruitment model, in which a PRC2-induced modification (H3K27me3) recruits PRC1. It has been demonstrated that PRC1 can be recruited in a manner independent of PRC2 and the H3K27me3 modification (46, 47). Interestingly, the PRC1 core component BMI1 was found at EBNA3C binding sites located in a regulatory element of both *COBLL1* and *ADAM28-ADAMDEC1*, whereas the PRC2 subunit SUZ12 was found only at the TSS of *COBLL1* (21). Other EBNA3C target genes have been shown to involve PcG proteins (42, 43, 48).

Recent studies have also shown that the composition of these PcG complexes is considerably more diverse than previously thought, particularly for PRC1. Noncanonical PRC1 complexes have been discovered that are capable of binding to chromatin and that function in the absence of PRC2 (49, 50). One important variant PRC1 complex, named PRC1.1, includes the KDM2B protein. KDM2B (also called FBXL10 and JHDM1B) is a histone lysine demethylase possessing the histone lysine demethylase catalytic domain JmjC, and it catalyzes demethylation of H3K4me3 (51, 52). Furthermore, it has been demonstrated that KDM2B is responsible for the recognition of nonmethylated CpG dinucleotides, leading to the recruitment of PRC1.1 to CpG islands of specific target genes (53–55).

In the present study, following the construction and use of novel EBNA3C recombinant EBVs, we demonstrated that EBNA3C uses its TFGC motif (HD motif) to epigenetically repress gene expression. Using the well-characterized EBNA3C target genes *COBLL1*, *ADAM28*, and *ADAMDEC1* as models, we found that EBNA3C HDmut was unable to fully remove the activation-associated chromatin mark H3K4me3 from the transcription start sites of its target genes. Further characterization revealed that the interaction between EBNA3C and KDM2B was important for the rapid loss of H3K4me3 and subsequent repression of EBNA3C target genes.

## RESULTS

### Generation and validation of LCLs with distinct EBNA3C mutant RBPJ interaction motifs.

**FIG 1:**
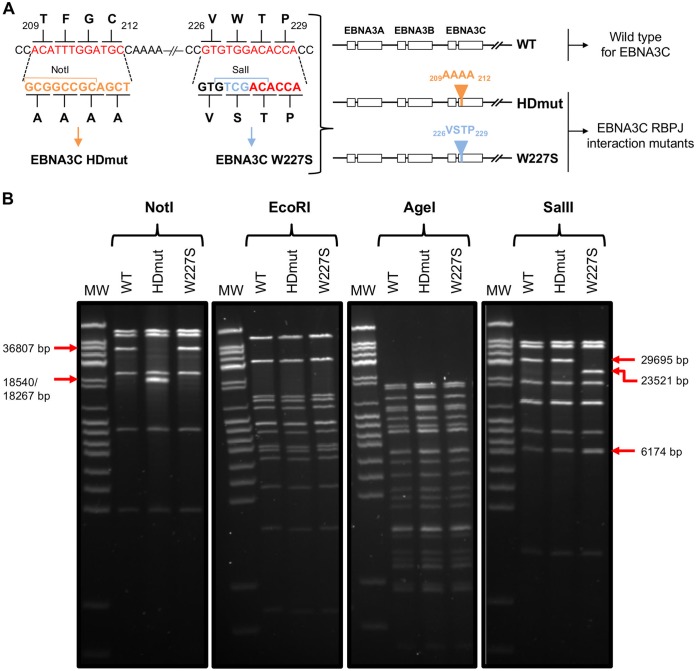
Generation of RBPJ interaction mutants of EBNA3C recombinant EBV BACs. (A) Schematic representation of mutations of the two RBPJ interaction motifs found in EBNA3C. The _209_TFGC_212_ and _226_VWTP_229_ motifs of EBNA3C were mutated to _209_AAAA_212_ and W227S, respectively. Mutations were introduced using an In-Fusion-based mutagenesis process, with the _209_AAAA_212_ mutation (homology domain [HD] mutant) introducing a NotI restriction site, whereas the W227S mutation introduced a SalI restriction site. The nucleotide sequence of wild-type (WT) EBNA3C is shown in red, whereas the HDmut and W227S sequences are shown in orange and blue, respectively. Both RBPJ interaction mutants of EBNA3C were introduced into the B95.8 EBV BAC by RecA-mediated homologous recombination. (B) BACs of WT and newly created RBPJ interaction mutant EBNA3C (HDmut and W227S) EBVs were analyzed by restriction digestion and pulsed-field gel electrophoresis. NotI restriction digestion showed that introduction of the _209_AAAA_212_ mutation created an additional NotI restriction site, cutting the 36,807-bp WT band into two bands, of 18,540 bp and 18,267 bp. EcoRI and AgeI restriction enzyme digestion revealed that the integrity of the EBNA3C mutant EBV BACs was maintained during the recombination process compared to that of the WT EBV BAC. SalI restriction digestion showed that introduction of the W227S mutation created an additional SalI restriction site, cutting the wild-type, 29,695-bp band into a 23,521-bp and a 6,174-bp band.

It was previously shown that mutations of both RBPJ interaction motifs (_209_AAAA_212_ and W227S mutations, giving a construct named EBNA3C RBPJ BM [21]) of EBNA3C gave an effective loss of RBPJ interaction as well as the disruption of EBNA3C transcriptional repression activity (21–24). To investigate further the effect of each single mutation on EBNA3C transcriptional activities, new EBV recombinants encoding each RBPJ interaction mutation of EBNA3C were constructed (EBNA3C HDmut and EBNA3C W227S) (Fig. 1A). EBNA3C HDmut contained well-known alanine substitution mutations, with _209_TFGC_212_ mutated to _209_AAAA_212_ (18, 22, 23), whereas EBNA3C W227S was based on the recently identified W227S mutation described by Calderwood and colleagues (24) (Fig. 1A). Restriction sites for NotI and SalI were introduced during the generation of both mutants to allow the recombinant virus genomes to be verified by restriction digestion and pulsed-field gel electrophoresis (Fig. 1A and B).

**FIG 2:**
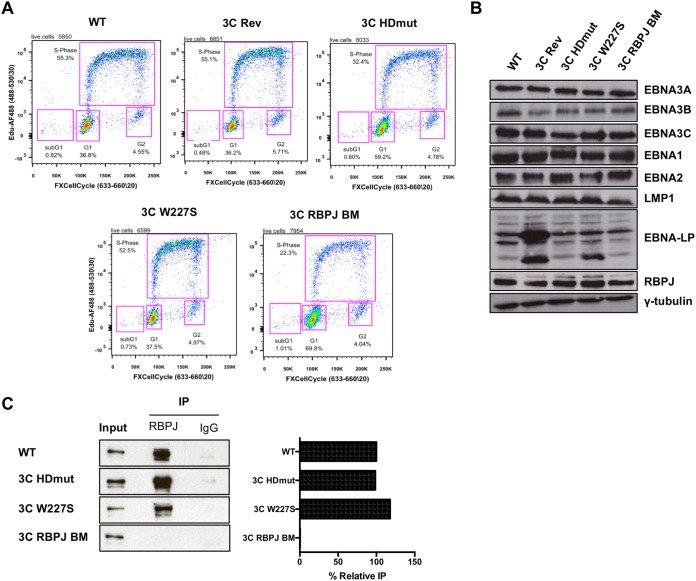
Validation of established EBNA3C HDmut and EBNA3C W227S LCLs. (A) Cell proliferation assay at day 36 after infection of primary B cells with the wild-type (WT), EBNA3C revertant (3C Rev), EBNA3C HDmut (3C HDmut), EBNA3C W227S (3C W227S), or EBNA3C RBPJ BM (3C RBPJ BM) recombinant virus. Live cells were analyzed for proliferation by measuring EdU incorporation and DNA content (by use of FxCycle Far Red DNA stain). Gates show the populations of cells in the sub-G_1_, G_1_, S, and G_2_/M phases. Data are representative of three independent infections. (B) Expression of Epstein-Barr virus latency-associated proteins EBNA3A, EBNA3B, EBNA3C, EBNA1, EBNA2, LMP1, and EBNA-LP, as well as RBPJ and γ-tubulin, was demonstrated by Western blotting of extracts of LCLs established from primary B cell infections with the wild-type (WT), EBNA3C revertant (3C Rev), EBNA3C HDmut (3C HDmut), EBNA3C W227S (3C W227S), and EBNA3C RBPJ BM (3C RBPJ BM) EBVs used for panel A. (C) Immunoprecipitation (IP) of RBPJ or an antibody isotype control (IgG) in the WT, 3C HDmut, 3C W227S, and 3C RBPJ BM LCLs and Western blotting of EBNA3C, as indicated. Input represents 10% of the lysate used in IPs. Pulldown of each EBNA3C mutant was quantified by use of ImageJ software, and nonspecific pulldown (IgG background) was subtracted. Each IP was normalized to its input and expressed as the percent relative IP compared to the positive-control level (EBNA3C WT).

The EBNA3C HDmut and EBNA3C W227S recombinant viruses were then used to infect primary CD19^+^ B cells and successfully led to the generation of LCLs. Cell proliferation, measured by the incorporation of 5-ethynyl-2′-deoxyuridine (EdU) 36 days after primary B cell infection, showed that around 50% of EBNA3C W227S cells were synthesizing DNA, which was the same as the level for cells infected with either the wild-type (WT) or EBNA3C revertant (3C Rev; considered WT) virus (Fig. 2A). Interestingly, the EdU incorporation assay demonstrated that the EBNA3C HDmut cells had reduced cell proliferation, as only 32% of EBNA3C HDmut cells were synthesizing DNA. As a negative control, an EBV recombinant encoding an RBPJ binding mutant of EBNA3C (3C RBPJ BM) was used to infect primary CD19^+^ B cells and showed that around 22% of RBPJ BM EBNA3C cells were synthesizing DNA. Western blot analyses of the EBNA3C W227S LCL showed levels of EBNA3 proteins as well as other EBV latency-associated proteins similar to those in the WT or EBNA3C revertant LCL (Fig. 2B). The EBNA3C level was slightly lower in the EBNA3C HDmut LCL than in the WT, and conversely, the EBNA2 level was slightly increased. This pattern of EBNA3C and EBNA2 expression was the same for the EBNA3C RBPJ BM LCL, as previously described (21). The level of RBPJ was unaffected in all LCLs. EBNA-LP frequently varies in level and repeat number as LCLs are established but is not considered relevant to EBNA3 function.

Finally, we investigated whether the newly generated EBNA3C mutants were impaired in the ability to interact with RBPJ. To do this, we performed co-IP assays with extracts of the WT LCL as well as LCLs 3C HDmut, 3C W227S, and 3C RBPJ BM (Fig. 2C). The co-IP assay showed that both the EBNA3C HDmut and W227S mutants retained interaction with RBPJ, while EBNA3C RBPJ BM did not.

### HD mutation impairs the transcriptional repression activity of EBNA3C in LCLs.

**FIG 3:**
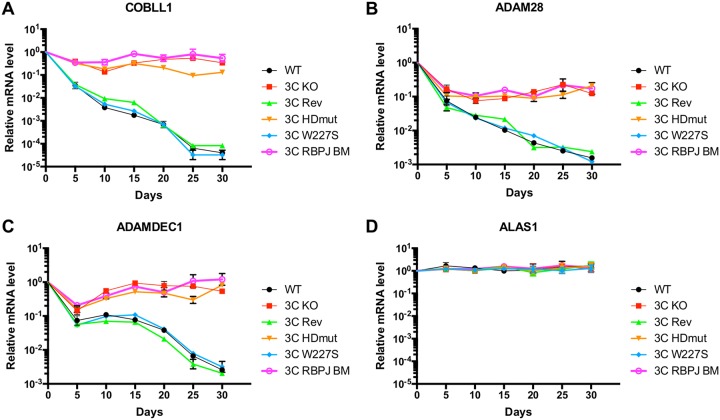
The TFGC motif of EBNA3C is important for its transcriptional repression activity. CD19^+^ purified B cells were infected with either the wild-type (WT), EBNA3C knockout (3C KO), EBNA3C revertant (3C Rev), EBNA3C HDmut (3C HDmut), EBNA3C W227S (3C W227S), or EBNA3C RBPJ BM (3C RBPJ BM) recombinant EBV and cultured for 30 days. RNA samples were taken at the indicated times after infection, and qPCR analysis performed on each. Gene expression of *COBLL1* (A), *ADAM28* (B), *ADAMDEC1* (C), and *ALAS1* (D) was normalized to that of the endogenous control *GNB2L1* and is shown relative to that in uninfected primary B cells. Data are representative of three independent time course experiments.

Because all LCLs established with the EBNA3C HDmut recombinant virus showed a reduction in cell proliferation compared to that of WT LCLs, we were interested in investigating whether this phenotype could be linked to an impairment of EBNA3C transcriptional activity during the infection and transformation of primary B cells by EBV. To do this, we set up a time course experiment to follow well-characterized EBNA3C target gene expression after infection of CD19^+^ B cells with different recombinant EBVs. Samples of cells were harvested at the time of infection and at 5-day intervals, and mRNA was extracted for analysis by reverse transcription-quantitative PCR (RT-qPCR). As expected, the EBNA3C knockout (3C KO) and EBNA3C RBPJ BM (3C RBPJ BM) viruses failed to regulate the *COBLL1* mRNA level, whereas EBNA3C-competent viruses (WT and 3C Rev) resulted in a rapid reduction of *COBLL1* gene expression over a period of 30 days after infection (Fig. 3A). Similar analysis of the EBNA3C W227S cells demonstrated that this mutant behaved like EBNA3C WT, leading to full repression of *COBLL1*. However, analysis of *COBLL1* expression in EBNA3C HDmut-infected cells showed only a small decrease of *COBLL1* mRNA compared to that in WT cells. The same effect was seen on two other well-known EBNA3C target genes, *ADAM28* (Fig. 3B) and *ADAMDEC1* (Fig. 3C). Relative expression levels of the control housekeeping gene *ALAS1* between the different infections were unaffected during the entire time course experiment (Fig. 3D). These results demonstrate that the transcriptional repression activities of EBNA3C are impaired by the HD mutation but not by the W227S mutation (considered WT for the rest of the study).

### EBNA3C HDmut binds RBPJ and recruits it to cellular target genes.

**FIG 4:**
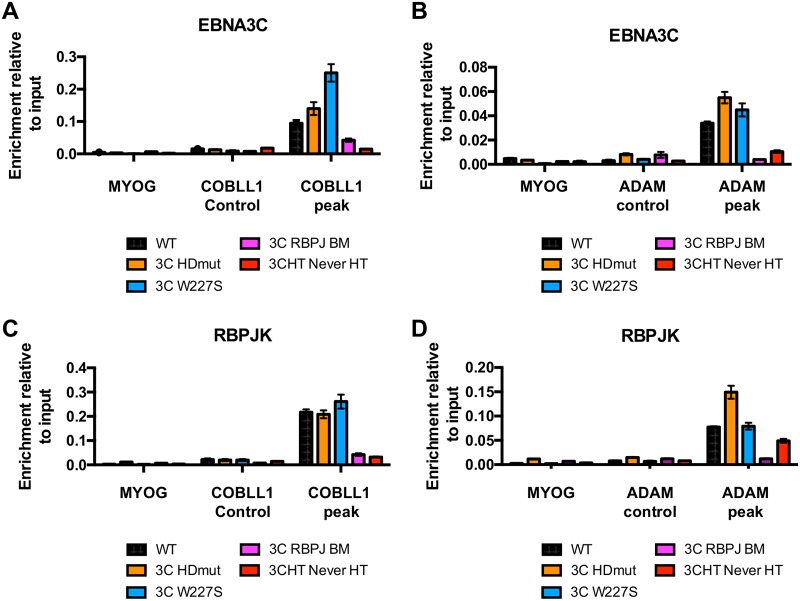
EBNA3C HDmut efficiently binds to RBPJ and recruits it to target genes. (A) ChIP-qPCR analyses using anti-EBNA3C to precipitate EBNA3C protein and chromatin associated with it from WT, EBNA3C HDmut, EBNA3C W227S, and EBNA3C RBPJ BM LCLs. As a control for antibody specificity, a similar ChIP was performed using the conditional LCL EBNA3C-HT never cultured with HT (LCL 3CHT Never HT). Primers for the Myogenin promoter (MYOG) as well as for a region inside the *COBLL1* genomic locus (*COBLL1* control) were used as negative controls for qPCR, whereas primers for the EBNA3C binding site at *COBLL1* (*COBLL1* peak) were used as positive controls for EBNA3C WT binding. ChIP values represent mean enrichment relative to the input level ± standard deviations for triplicate qPCRs for the ChIP and input of each sample. (B) Same as panel A, but using control primers for the *ADAM* cluster region (*ADAM* control) as well as primers for the *ADAM28-ADAMDEC1* intergenic enhancer (*ADAM* peak). (C) Same as panel A, but using anti-RBPJ antibody for ChIP analyses. (D) Same as panel B, but using anti-RBPJ antibody for ChIP analyses.

To understand the molecular mechanisms behind the failure of EBNA3C HDmut to fully repress transcription, we assessed whether the HD mutation had any effect on the binding of EBNA3C to its target genes. Anti-EBNA3C chromatin immunoprecipitation (ChIP) assays were performed on LCLs WT, 3C HDmut, 3C W227S, and 3C RBPJ BM, followed by qPCR to determine the levels of EBNA3C protein bound to the *COBLL1* peak and the *ADAM* peak, previously identified as EBNA3C binding sites on *COBLL1* and at the *ADAM28-ADAMDEC1* genomic locus, respectively (21). We found that EBNA3C HDmut, though expressed less in LCL 3C HDmut than in LCL WT (Fig. 2B), was still recruited to both the *COBLL1* and *ADAM* peaks, as efficiently as or even more efficiently than the recruitment of EBNA3C WT (Fig. 4A and B). Interestingly, EBNA3C W227S was found to bind to the *COBLL1* and *ADAM* peaks more efficiently than EBNA3C WT did. A drastic reduction of EBNA3C binding was also found on both genes in the 3C RBPJ BM cell line, further supporting the dynamic recruitment/stabilization model of RBPJ-EBNA3C complexes under repression. As expected, no EBNA3C binding was observed in an EBNA3C conditional LCL (p16 null background) (12) that had never been cultured with HT (LCL 3CHT Never HT). Furthermore, no binding was observed using control primer pairs covering a control region on the *COBLL1* locus, the *ADAM28-ADAMDEC1* locus, or the Myogenin promoter (*MYOG*). Furthermore, EBNA3C HDmut and EBNA3C W227S recruited RBPJ to the EBNA3C peak on both the *COBLL1* (Fig. 4C) and *ADAM* (Fig. 4D) genes, confirming that both mutants still interacted with RBPJ at levels comparable to or higher than that with EBNA3C WT.

### Recruitment of PcG proteins to EBNA3C target genes is not impaired by HD mutation.

In the last few years, it has emerged that EBNA3C-mediated gene silencing often involves the recruitment of Polycomb repressive complexes 1 and 2 (PRC1 and PRC2) to target genes, leading to deposition of the repressive mark H3K27me3. Notably, it has clearly been shown that the PRC1 family member BMI1 and the PRC2 component SUZ12 are recruited to the *COBLL1* genomic locus by EBNA3C at its binding site (*COBLL1* peak) and its transcription start site (*COBLL1* TSS), respectively. It is unclear whether BMI1 and SUZ12 are recruited by direct interaction with EBNA3C or if this represents a default mechanism of *COBLL1* repression. The EBNA3C-mediated regulation of the *ADAM28-ADAMDEC1* locus is different, as BMI1 recruitment increases at the *ADAM* peak in the first few weeks of repression and then quickly decreases, reaching the same start level as that found when EBNA3C is inactive (21). Furthermore, it has been reported that no recruitment of SUZ12 was detected across the *ADAM28-ADAMDEC1* locus, leading to the deposition of a low level of H3K27me3 (21).

**FIG 5:**
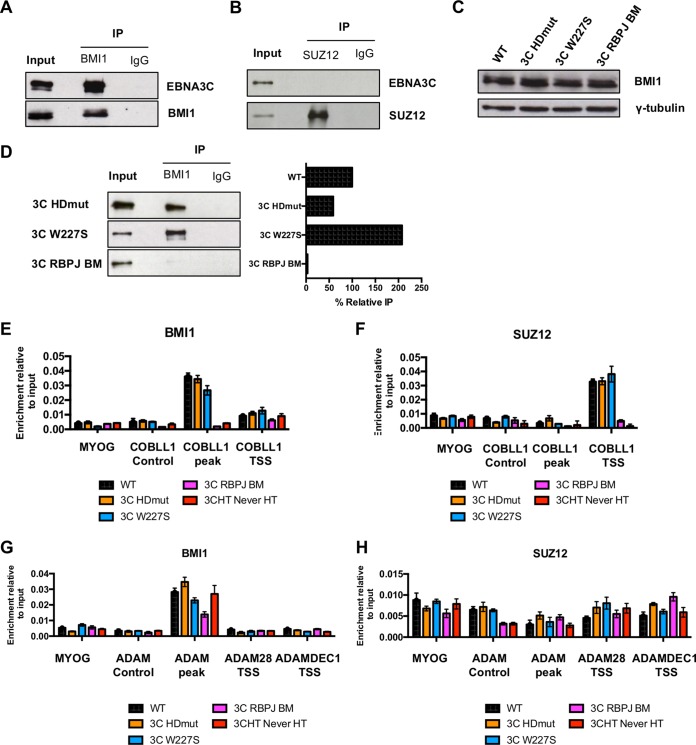
EBNA3C HDmut still recruits Polycomb proteins to target genes. (A and B) BMI1 and SUZ12 were immunoprecipitated (IP) from a WT LCL protein extract by use of anti-BMI1 (A), anti-SUZ12 (B), or an antibody isotype control (IgG). Immunoprecipitated and coimmunoprecipitated proteins were analyzed by Western blotting. Input corresponds to 10% of the cell extract used for IPs. (C) BMI1 and γ-tubulin protein expression in LCLs WT, EBNA3C HDmut, EBNA3C W227S, and EBNA3C RBPJ BM. (D) Immunoprecipitation performed with anti-BMI1 or an antibody isotype control (IgG) on extracts of the 3C HDmut, 3C W227S, and 3C RBPJ BM LCLs and Western blotting of EBNA3C, as indicated. Input represents 10% of the lysate used in IPs. Pulldown of each EBNA3C mutant was quantified by use of ImageJ software, and nonspecific pulldown (IgG background) was subtracted. Each IP was normalized to its input and expressed as the percent relative IP compared to the positive-control level (EBNA3C WT; see panel A). (E and F) ChIP was performed for BMI1 (E) and SUZ12 (F) within the *COBLL1* locus on extracts of the WT, EBNA3C HDmut, EBNA3C W227S, and EBNA3C RBPJ BM LCLs as well as the LCL EBNA3C-HT Never HT. A primer pair for the Myogenin promoter (MYOG) was used as a negative control. ChIP values represent enrichment relative to the input level ± standard deviations for triplicate qPCRs for the ChIP and input of each sample. (G and H) Same as panels E and F, but using primers across the *ADAM28-ADAMDEC1* locus.

In order to determine whether EBNA3C could be found in complex with BMI1 or SUZ12, coimmunoprecipitation assays were performed with LCLs. Interestingly, immunoprecipitation of BMI1 by use of an anti-BMI1 antibody led to EBNA3C being coimmunoprecipitated strongly in LCL WT extracts (Fig. 5A). However, we were unable to find EBNA3C in complex with SUZ12 by this method (Fig. 5B). Next, we investigated whether the loss of gene repression activity displayed by EBNA3C HDmut could be due to an impairment in its interaction with BMI1 or to a decrease of the BMI1 protein level in LCLs. Analysis of protein levels by Western blotting demonstrated that BMI1 protein levels were equal among the WT, EBNA3C HDmut, EBNA3C W227S, and EBNA3C RBPJ BM cell lines (Fig. 5C). Co-IP analyses showed that whereas EBNA3C W227S interacted with BMI1 more efficiently than EBNA3C WT did, EBNA3C HDmut still interacted with BMI1, but to a lesser extent (60%) (Fig. 5D). EBNA3C RBPJ BM, however, did not interact with BMI1. Interestingly, the apparently decreased interaction between EBNA3C HDmut and BMI1 did not affect BMI1 recruitment to *COBLL1*, as ChIP-qPCR experiments showed that BMI1 was efficiently recruited to the *COBLL1* peak in the EBNA3C HDmut LCL (Fig. 5E). Furthermore, even though no interaction between EBNA3C and SUZ12 was discovered, SUZ12 was still found to be enriched at the *COBLL1* TSS, suggesting that this recruitment is indirect and part of a default mechanism of gene repression (Fig. 5F). Interestingly, the same pattern for BMI1 recruitment was found on the *ADAM28-ADAMDEC1* locus, where no difference in BMI1 level was detected between LCLs WT and 3C HDmut at the *ADAM* peak (Fig. 5G). Furthermore, and as expected, no SUZ12 recruitment was detected across the whole locus (Fig. 5H).

Taken together, these data demonstrate that even though EBNA3C HDmut fails to fully repress *COBLL1*, *ADAM28*, and *ADAMDEC1*, this mutant behaves like EBNA3C WT in the recruitment of the PcG proteins to its target genes.

### Removal of the histone activation mark H3K4me3 is impaired in EBNA3C HDmut.

**FIG 6:**
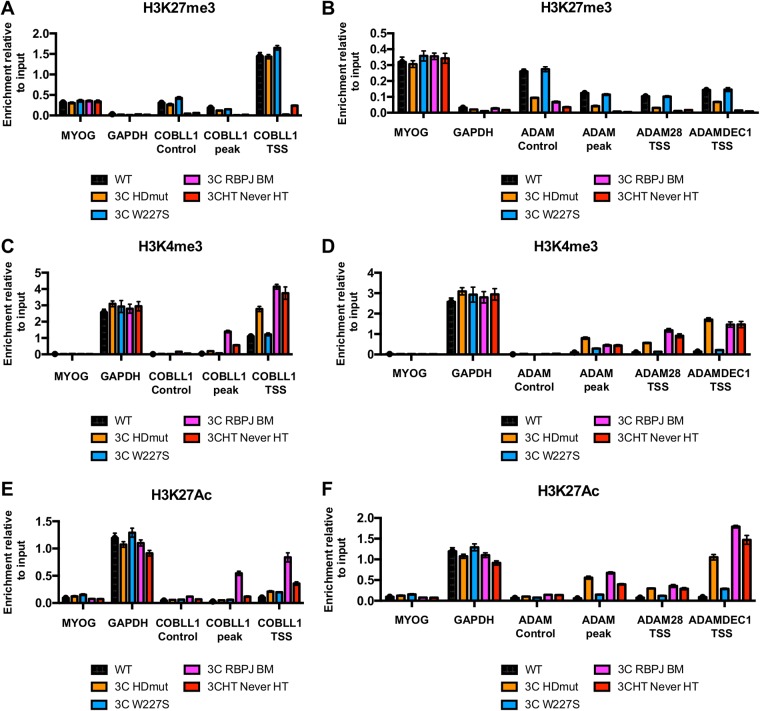
Epigenetic changes at sites within the *COBLL1* and *ADAM28-ADAMDEC1* loci in EBNA3C mutant LCLs. (A) ChIP for H3K27me3 on the WT, EBNA3C HDmut, EBNA3C W227S, and EBNA3C RBPJ BM LCLs at locations across the *COBLL1* locus, at the Myogenin promoter (MYOG), or at the *GAPDH* promoter, as indicated. As a control, a similar ChIP was performed using the conditional LCL 3CHT Never HT. ChIP values represent enrichment relative to the input level ± standard deviations for triplicate qPCRs for the ChIP and input of each sample. (B) Same as panel A, but using primers across the *ADAM28-ADAMDEC1* locus. (C and D) Same as panels A and B, respectively, but using an anti-H3K4me3 antibody for ChIP analyses. (E and F) Same as panels A and B, respectively, but using an anti-H3K27Ac antibody for ChIP analyses.

Because EBNA3C-mediated gene repression has been shown to correlate with a high level of the repressive histone mark H3K27me3 (catalyzed by PRC2), it was essential to verify whether this repressive mark was found around the *COBLL1*, *ADAM28*, and *ADAMDEC1* TSS in LCL EBNA3C HDmut, in which these genes are still expressed. To do that, ChIP-qPCR analyses were performed with LCLs and an anti-H3K27me3 antibody. The H3K27me3 level was high across LCLs WT, 3C HDmut, and 3C W227S at the *COBLL1* TSS compared to those in negative-control LCLs 3C RBPJ BM and 3CHT Never HT and was therefore not affected by EBNA3C HDmut (Fig. 6A). It is important that LCL EBNA3C W227S (considered WT) exhibited a slightly higher level of the repressive mark H3K27me3 at the *COBLL1* TSS than those in LCLs EBNA3C WT and 3C HDmut. When it was investigated across the *ADAM28-ADAMDEC1* locus, the repressive mark H3K27me3 was found to be increased in LCLs WT and 3C W227S compared to its level in the negative-control LCLs EBNA3C RBPJ BM and 3CHT Never HT (Fig. 6B). Interestingly, the H3K27me3 level found in LCL 3C HDmut was lower than that in LCL WT but was always higher than the H3K27me3 level found in negative-control LCLs (3C RBPJ BM and 3CHT Never HT). As expected, the level of H3K27me3 across the *ADAM28-ADAMDEC1* locus was low compared to the level found at the *COBLL1* TSS (compare Fig. 6A and B).

Gene repression by EBNA3C has been shown to take place in two steps. First, initial repression is associated with a loss of activation-associated histone marks at the EBNA3C target gene TSS, resulting in a decrease of mRNA transcription. Subsequent deposition of repressive histone marks leads to a fully repressed state of the target gene. After examining the H3K27me3 level, we next decided to assess the level of the other trimethylation mark, H3K4me3, a chromatin modification well known to be associated with active gene promoters and shown to be reduced in target genes repressed by EBNA3C or EBNA3A (21, 56). As expected, ChIP analysis of LCLs EBNA3C WT and EBNA3C W227S (in which *COBLL1* expression is repressed) revealed a loss of H3K4me3 at the *COBLL1* TSS compared to the high H3K4me3 enrichment in the positive-control LCLs RBPJ BM and 3CHT Never HT, in which *COBLL1* is expressed (Fig. 6C). The same analysis revealed that H3K4me3 was enriched at the *COBLL1* TSS in LCL EBNA3C HDmut compared to that in either LCL 3C WT or 3C W227S. Interestingly, ChIP analysis revealed the same pattern of H3K4 trimethylation for the *ADAM28-ADAMDEC1* locus, with higher H3K4me3 levels found at both the *ADAM28* and *ADAMDEC1* TSS in LCL EBNA3C HDmut than at those in LCLs EBNA3C WT and EBNA3C W227S (Fig. 6D).

Finally, we assessed the level of another activation mark, H3K27Ac, also demonstrated to be reduced at the TSS of EBNA3C target genes. As expected, ChIP experiments revealed a higher H3K27Ac level at the *COBLL1* TSS (Fig. 6E), the *ADAM28* TSS, and the *ADAMDEC1* TSS (Fig. 6F) in the positive-control cell lines (LCLs 3C RBPJ BM and 3CHT Never HT) than in LCL WT. Interestingly, no difference in the activation mark H3K27Ac was found between LCLs 3C HDmut and 3C W227S at the *COBLL1* TSS, where H3K27Ac levels were slightly higher than the level found in LCL WT (Fig. 6E). However, for the *ADAM28-ADAMDEC1* locus, ChIP analysis revealed a higher level of H3K27Ac on both the *ADAM28* and *ADAMDEC1* TSS in LCL 3C HDmut than on those in LCLs WT and 3C W227S, suggesting that EBNA3C HDmut is impaired in the removal of the activation mark H3K27Ac at the *ADAM28-ADAMDEC1* locus.

Taken together, these analyses showed that EBNA3C HDmut is consistently impaired in the ability to fully remove the activation mark H3K4me3 on EBNA3C target genes *COBLL1*, *ADAM28*, and *ADAMDEC1*.

### Expression of EBNA3C HDmut in LCL 3CHT Never HT fails to fully repress EBNA3C target genes.

**FIG 7:**
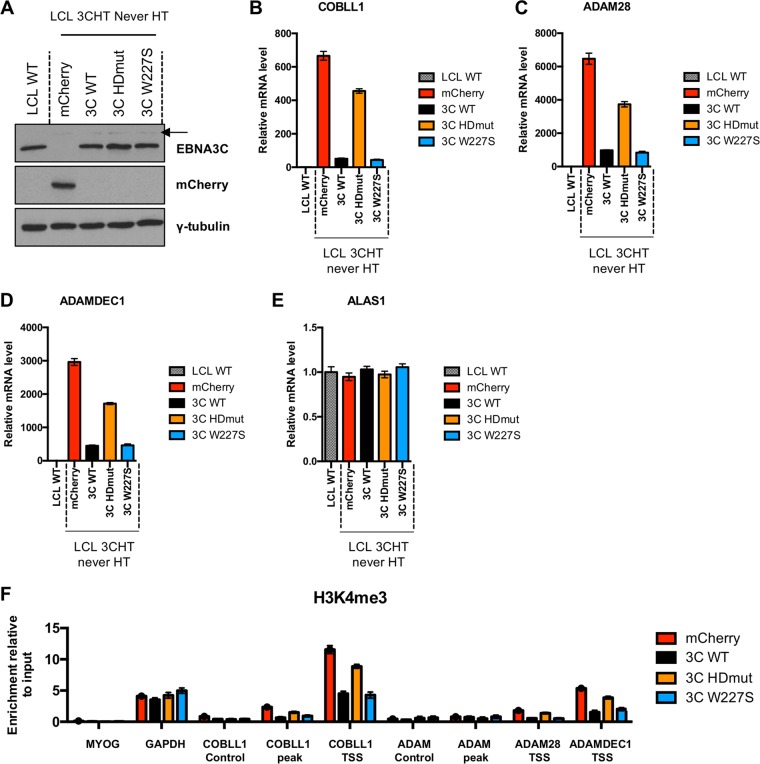
*COBLL1*, *ADAM28*, and *ADAMDEC1* regulation in EBNA3C lentiviral expression system in LCLs. (A) EBNA3C, mCherry, and γ-tubulin protein expression in the LCL 3CHT Never HT infected with lentiviruses expressing either mCherry, EBNA3C WT, EBNA3C HDmut, or EBNA3C W227S for 30 days. A protein extract of LCL WT was used as a positive control for EBNA3C expression. The residual expression of EBNA3C-HT is shown by a black arrow. (B to E) Expression levels of *COBLL1* (B), *ADAM28* (C), *ADAMDEC1* (D), and *ALAS1* (E) were determined for the LCLs used for panel A. (F) The H3K4me3 level was assessed by ChIP assay of LCLs infected with the lentiviruses used for panel A. Primer pairs across the *COBLL1* and *ADAM28-ADAMDEC1* loci were used, as well as primers for Myogenin (MYOG) and *GAPDH*, as negative and positive controls, respectively. ChIP values represent enrichment relative to the input level ± standard deviations for triplicate qPCRs for the ChIP and input of each sample. Data are representative of one of at least two independent experiments.

To further demonstrate that EBNA3C HDmut fails to fully remove the activation mark H3K4me3 on *COBLL1*, *ADAM28*, and *ADAMDEC1* in LCLs, we made use of a lentiviral vector system. Lentiviruses carrying cDNA encoding mCherry (used as a negative control) or cDNA encoding EBNA3C WT, EBNA3C HDmut, or EBNA3C W227S were produced and used to infect LCL 3CHT Never HT. Analysis of protein levels by Western blotting showed that the levels of EBNA3C WT and EBNA3C mutants were similar to the level of EBNA3C found in LCL WT (Fig. 7A). The level of the nonfunctional protein EBNA3C-HT was barely detectable in LCL 3CHT Never HT, due to its sequestration and degradation in the cytoplasm, consistent with previous studies (15, 21). As previously identified in Fig. 3, only the expression of EBNA3C HDmut led to a failure to fully decrease *COBLL1* mRNA (compared to that in mCherry control cells), whereas expression of both EBNA3C WT and EBNA3C W227S induced a decrease in *COBLL1* expression (Fig. 7B). The same pattern of regulation was detected for the *ADAM28* (Fig. 7C) and *ADAMDEC1* (Fig. 7D) mRNA levels. As expected, the mRNA level of the control housekeeping gene *ALAS1* showed no change after infection with any of the lentiviruses used (Fig. 7E). Finally, ChIP-qPCR analyses were performed to assess the level of H3K4me3 across EBNA3C target genes in those cells. Interestingly, only EBNA3C HDmut did not fully remove H3K4me3 marks at the *COBLL1* TSS, the *ADAM28* TSS, and the *ADAMDEC1* TSS (Fig. 7F).

These results indicate that the failure of EBNA3C HDmut to repress the target genes *COBLL1*, *ADAM28*, and *ADAMDEC1* may be due in part to an impairment in the removal of the activation mark H3K4me3 at the respective transcription start sites.

### EBNA3C requires its TFGC motif to interact with KDM2B.

**FIG 8:**
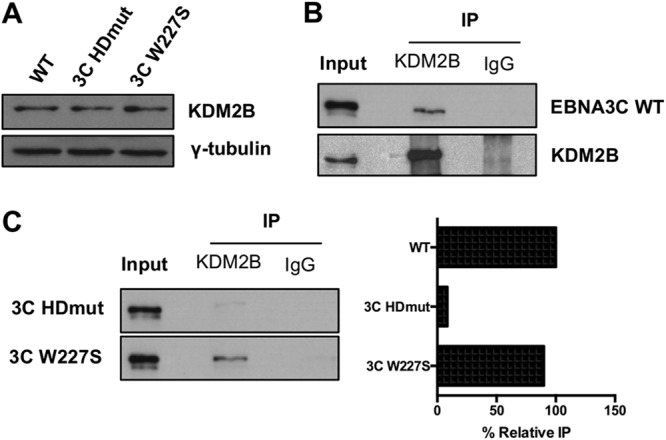
The EBNA3C protein interacts with the histone demethylase KDM2B. (A) KDM2B and γ-tubulin protein expression in LCLs WT, EBNA3C HDmut, and EBNA3C W227S. (B) Immunoprecipitation (IP) of KDM2B or an antibody isotype control (IgG) in the LCL EBNA3C WT used for panel A and Western blotting of EBNA3C and KDM2B, as indicated. Input represents 10% of the lysate used for IP. (C) Immunoprecipitation of KDM2B or an antibody isotype control (IgG) in the LCLs EBNA3C HDmut and EBNA3C W227S used for panel A and Western blotting of EBNA3C, as indicated. Input represents 10% of the lysate used in IPs. Pulldown of each EBNA3C mutant was quantified by use of ImageJ software, and nonspecific pulldown (IgG background) was subtracted. Each IP was normalized to its input and expressed as the percent relative IP compared to the positive-control level (EBNA3C WT; see panel B).

We next investigated the molecular mechanism behind the removal of H3K4me3 marks on EBNA3C target genes. KDM2B is a histone lysine demethylase that has been shown to catalyze the removal of the trimethyl mark from H3K4me3. The KDM2B protein levels in LCL WT (EBNA3C WT), LCL EBNA3C HDmut, and LCL EBNA3C W227S were measured by Western blotting, and KDM2B was expressed at the same level across the different LCLs (Fig. 8A). An anti-KDM2B antibody was then used for coimmunoprecipitation assays to test whether any physical interaction between EBNA3C and KDM2B could be found in LCLs (Fig. 8B). Interestingly, EBNA3C WT was successfully coimmunoprecipitated with KDM2B. We then tested whether EBNA3C HDmut, which is impaired in the capacity to fully remove the H3K4me3 mark from its target genes, and EBNA3C W227S were still able to interact with the demethylase KDM2B. Interestingly, immunoprecipitation with an anti-KDM2B antibody showed that whereas the EBNA3C W227S mutant still interacted with KDM2B, though to a lesser extent than that with the EBNA3C WT control (80%) (Fig. 8C), EBNA3C HDmut showed a great impairment in the capacity to coimmunoprecipitate with KDM2B (8%).

### shRNA-mediated depletion of KDM2B in LCLs shows that it is an important factor for EBNA3C-mediated gene repression.

**FIG 9:**
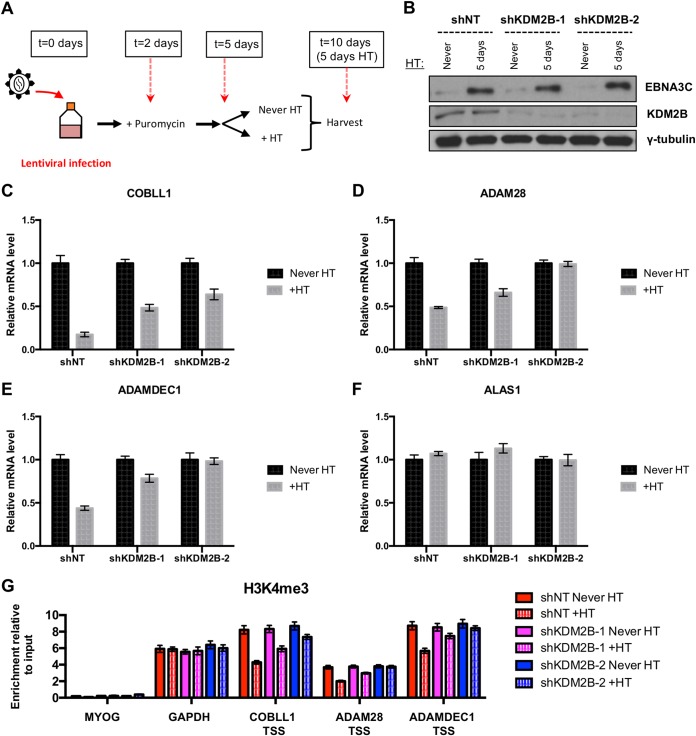
KDM2B is required for efficient repression of *COBLL1*, *ADAM28*, and *ADAMDEC1* by EBNA3C. (A) Schematic of time course experiment. LCL 3CHT Never HT cells were infected with lentiviruses carrying either a control nontargeting (NT) shRNA or shRNAs directed against KDM2B. After 2 days, puromycin was added to the culture medium to select the infected cells. HT was then added to half of the culture (+HT), and the other half was left without HT (Never HT). After 5 days of HT treatment, cells were harvested. (B) Western blots of extracts from the time course experiment described for panel A, showing efficient knockdown of KDM2B after infection with lentiviruses carrying two different shRNAs against KDM2B (shKDM2B-1 and shKDM2B-2) and showing stabilization of EBNA3C after addition of HT. (C to F) Expression levels of *COBLL1* (C), *ADAM28* (D), *ADAMDEC1* (E), and *ALAS1* (F) were measured by qPCR with the cells used for panel B. Gene expression was normalized to that of the endogenous control *GNB2L1* and is shown relative to that of each cell line never treated with HT (Never HT). (G) The H3K4me3 level was assessed by ChIP assay of the LCLs 3CHT shNT, 3CHT shKDM2B-1, and 3CHT shKDM2B-2 used for panel B. ChIP values represent enrichment relative to the input level ± standard deviations for triplicate qPCRs for the ChIP and input of each sample. Data are representative of one of two independent infections.

The EBNA3C-mediated silencing of *COBLL1*, *ADAM28*, and *ADAMDEC1* is rapid, and it was previously shown that activation of EBNA3C in the conditional LCL 3CHT resulted in a reduction of *COBLL1*, *ADAM28*, and *ADAMDEC1* mRNAs (with the *COBLL1* reduction being more drastic than those of *ADAM28* and *ADAMDEC1*) after only 5 days of HT addition to the culture medium (15, 21). Because we had shown that the demethylase KDM2B interacts with EBNA3C, we then used this conditional EBNA3C cell line to assess the potential role of KDM2B in the repression of its well-characterized target genes. Lentiviruses carrying a control nontargeting (NT) small hairpin RNA (shRNA) or shRNAs specific for KDM2B were used to infect the conditional LCL 3CHT Never HT for 2 days before addition of puromycin to the cell culture (Fig. 9A). After 3 days of selection, the cells were passaged, and half of the culture was left without HT (Never HT), while HT was added to the other half (+HT) for 5 days. The cells were then harvested for protein, RNA, and ChIP analyses. Analysis by Western blotting confirmed that both lentiviruses carrying shRNA against KDM2B (shKDM2B-1 and shKDM2B-2) efficiently depleted their target compared to that in the same cell line infected with lentiviruses expressing a nontargeting shRNA (shNT) (Fig. 9B). As expected, EBNA3C was shown to be stabilized after addition of HT to the culture medium. Interestingly, EBNA3C-mediated *COBLL1* repression was less pronounced when KDM2B was knocked down (Fig. 9C). *ADAM28* (Fig. 9D) and *ADAMDEC1* (Fig. 9E) gene repression was also reduced by the knockdown of KDM2B, and even prevented in the case of shKDM2B-2. The relative expression of the control housekeeping gene *ALAS1* was unaffected by the knockdown of KDM2B and the activation of EBNA3C (Fig. 9F).

Finally, to assess the effect of KDM2B on the H3K4me3 level in LCLs, we performed a ChIP analysis using the LCLs 3CHT shNT, shKDM2B-1, and shKDM2B-2 left without HT (Never HT) or treated with HT for 5 days (Fig. 9G). As expected, the activation of EBNA3C by the addition of HT resulted in a reduction of the H3K4me3 levels at the TSS of *COBLL1*, *ADAM28*, and *ADAMDEC1* in LCL 3CHT shNT. However, this reduction in H3K4me3 level was less pronounced in both shKDM2B cell lines (shKDM2B-1 and shKDM2B-2) treated with HT.

Taken together, these results show, for the first time, that KDM2B is important for the removal of H3K4me3 from the *COBLL1*, *ADAM28*, and *ADAMDEC1* TSS and that KDM2B is needed for efficient repression of *COBLL1*, *ADAM28*, and *ADAMDEC1* by EBNA3C.

## DISCUSSION

Although it is now well established that the Epstein-Barr virus protein EBNA3C is essential for transformation of primary B cells and for repression of host tumor suppressor genes, the precise molecular mechanisms by which EBNA3C silences cellular target genes are still poorly understood. In the present study, by using novel EBNA3C recombinant EBVs encoding EBNA3C with mutations in two previously described motifs important for RBPJ interaction, we explored the molecular mechanisms by which EBNA3C represses cellular gene expression. This revealed that the TFGC motif, located in the N-terminal homology domain of EBNA3C, is important for transcriptional repression of EBNA3C's cellular target genes. While EBNA3C HDmut (TFGC_212_ to AAAA_212_) was still able to recruit PcG proteins, as EBNA3C WT can, this mutant was found to be impaired in the capacity to efficiently remove the activation-associated chromatin mark H3K4me3 from its target genes.

RBPJ is a cellular transcription factor that plays an important function in EBV biology, as shown by its interaction with all members of the EBNA3 family of proteins as well as with the EBV transactivator EBNA2 (10). Depletion of RBPJ in LCLs by use of shRNA has also been shown to decrease cell viability (57), suggesting a crucial role for this protein in LCL proliferation and survival. Two different motifs within EBNA3C have been identified to be important for RBPJ interaction, and it is known that mutation of both leads to a loss of interaction and loss of EBNA3C transcriptional repression function. To discover whether a single motif mutation could also affect EBNA3C repressive function in the context of EBV infection, we constructed two novel EBV bacterial artificial chromosome (BAC) recombinants, expressing EBNA3C HDmut or EBNA3C W227S. Infection of primary B cells with these mutants led to the establishment of LCLs. However, the LCL EBNA3C HDmut exhibited a decrease in the number of proliferating cells compared to that for LCL WT or LCL EBNA3C W227S, suggesting that the HD mutation leads to an impairment in EBNA3C functions. Interestingly, time course analysis of EBNA3C target gene expression during the primary B cell infection showed that whereas EBNA3C W227S repressed *COBLL1*, *ADAM28*, and *ADAMDEC1* as efficiently as EBNA3C WT did, EBNA3C HDmut was partially deficient in its transcriptional repression functions. This effect was independent of RBPJ interaction, as EBNA3C HDmut still interacted with RBPJ and recruited it to chromatin at target genes.

These findings help to clarify the role in EBNA3C function played by the TFGC motif. Previous studies demonstrated the essential requirement of this motif in both gene regulation and sustained LCL proliferation in *trans* (18, 22, 23). However, this has been confounded by the mapping of the EBNA3C interaction with RBPJ to the EBNA3 homology domain. The results described in our study are in agreement with others (21, 24) showing that loss of the TFGC motif alone does not prevent the formation of an RBPJ-EBNA3C complex, nor does it prevent EBNA3C-directed recruitment of RBPJ to target loci (22, 23). Furthermore, we also found that EBNA3C HDmut LCLs compared to EBNA3C WT LCLs are impaired in the ability to regulate host target genes, as previously demonstrated for *TCL1* (23). It is important that we consistently observed establishment of HDmut LCLs when primary B cells were infected with EBNA3C HDmut virus, whereas it was shown that transcomplementation of LCL EBNA3C-HT with EBNA3C HDmut failed to maintain cell proliferation (22, 23). This discrepancy might be the result of the different selection pressures exerted in the two different systems, with established LCLs being intolerant of changes to EBNA3C functionality. Most importantly, our study demonstrated that the TFGC motif plays a more fundamental role in EBNA3C function than just the interaction with RBPJ. The majority of EBNA3C interaction partners have been mapped to the homology domain, with KDM2B being no exception, suggesting a vital role in complex formation (10). Furthermore, comparative analysis of colocalization between EBNA3C and RBPJ by ChIP sequencing (ChIP-seq) suggested that RBPJ is not the dominant determinant factor in EBNA3C binding to chromatin (14, 15, 37, 58). These studies called into question the importance of forming an RBPJ-EBNA3C complex, with other homology domain-based interactions far more likely to be critical to EBNA3C function. Further studies to reassess the EBNA3C-RBPJ interaction are needed to precisely map the significant residues and motifs critical to formation of this complex. It has been shown that additional residues, besides the ΦWΦP motif within the N terminus of the Notch RAM domain, significantly contribute to the binding of RBPJ (25, 26, 29, 30, 32, 59). For EBNA3C, these important residues include the TFGC motif and might also include closer residues downstream or upstream of the VWTP motif.

The use of the HD mutant offered us the unique opportunity to explore the mechanisms of EBNA3C gene repression in an RBPJ-independent context. A two-step epigenetic mechanism for EBNA3C-mediated gene repression, involving different histone modifications, has been proposed (21). It has been demonstrated that repression of *COBLL1* involves a rapid loss of active histone marks (H3K27Ac and H3K4me3) at its TSS followed by the deposition of a high level of the repressive mark H3K27me3 (histone 3 lysine 27 trimethylation), catalyzed by PRC2. Because EBNA3C-mediated gene repression at *COBLL1* is known to involve the Polycomb protein complexes PRC1 and PRC2, we first decided to investigate whether EBNA3C HDmut was deficient in the recruitment of both complexes to *COBLL1*. EBNA3C HDmut still recruited BMI1 and SUZ12 to the *COBLL1* peak and TSS, respectively. However, we found that whereas EBNA3C could interact with BMI1, EBNA3C could not be found in complex with the PRC2 component SUZ12. This result strongly suggests that the PRC2 complex is recruited indirectly to *COBLL1* by EBNA3C, most likely via interaction with the PRC1 complex. It is known that EBNA3C mediates regulation of transcription by long-distance chromatin interactions (chromosome “looping”) between promoter and enhancer elements. Furthermore, Polycomb complexes have been shown to mediate the formation of higher-order chromosome structures (60–62). Even though it has not been tested formally, it is tempting to speculate that EBNA3C interaction with PRC1 might recruit PRC2, leading to the creation of chromatin looping and subsequent regulation of gene repression. Trimethylation of lysine 27 of histone 3 (H3K27me3) was induced by EBNA3C at the *COBLL1* genomic locus regardless of the mutant expressed, confirming the efficient recruitment of the Polycomb proteins. The repression of the *ADAM28-ADAMDEC1* locus also involved a loss of active histone marks at both gene TSS, followed by an increase of the H3K27me3 level. However, in contrast to what was observed at the *COBLL1* TSS, the deposition of H3K27me3 at the *ADAM28* TSS and the *ADAMDEC1* TSS reached lower levels, and no SUZ12 recruitment could be detected at both TSS. Interestingly, whereas the recruitment of BMI1 to the *ADAM28-ADAMDEC1* locus was not affected by the EBNA3C HD mutation, the deposition of the repressive mark H3K27me3 was found to be impaired by this mutant.

Analysis of the H3K27ac level revealed a different pattern depending on the target gene investigated. The active acetylation mark level was found at a low level on the *COBLL1* TSS, and the levels were comparable between LCLs EBNA3C HDmut and EBNA3C W227S. However, the H3K27Ac level was found to be high in LCL EBNA3C HDmut across the *ADAM28-ADAMDEC1* locus, suggesting that the HD mutant is impaired in the removal of the acetylation mark. These results suggest that the mechanism involved in the removal of H3K27Ac might be different between *COBLL1* and *ADAM28-ADAMDEC1*. Interestingly, it has been shown that EBNA3C interacts with HDAC1 through the TFGC motif (63). However, the relationship between this interaction and the acetylation level on EBNA3C target genes has yet to be determined. It is important that the high level of H3K27Ac found at both TSS (those of *ADAM28* and *ADAMDEC1*) in LCL EBNA3C HDmut might explain why the level of H3K27me3 is impaired in this cell line. Indeed, both chromatin modifications (acetylation and trimethylation) take place at the same lysine residue on histone H3. It has been shown that H3K27Ac and H3K27me3 are mutually exclusive and that H3K27Ac prevents trimethylation of H3K27 at sites in Polycomb target genes (64).

Analysis of H3K4me3 in LCL EBNA3C HDmut revealed a relatively high level of this active mark around the TSS of the EBNA3C target genes *COBLL1*, *ADAM28*, and *ADAMDEC1* compared to those in LCLs EBNA3C WT and EBNA3C W227S. This was further confirmed using lentiviral vectors allowing the expression of EBNA3C WT and EBNA3C HDmut in an LCL deficient for functional EBNA3C (LCL 3CHT Never HT). EBNA3C HDmut expression failed to fully repress *COBLL1* as well as *ADAM28* and *ADAMDEC1* and did not completely remove the activation-associated H3K4me3 mark on their transcription start sites. Interestingly, we next found that KDM2B, a protein implicated in the demethylation of H3K4me3, interacts with EBNA3C. This interaction was greatly impaired when the TFGC motif of EBNA3C was mutated (HDmut). Finally, to determine whether KDM2B was important for EBNA3C-mediated repression of its target genes, we made use of the conditional LCL 3CHT Never HT combined with lentiviruses carrying shRNA to knock down the expression of KDM2B. We showed that KDM2B was important for the loss of the H3K4me3 mark as well as for the repression of EBNA3C target genes. We speculate that EBNA3C may recruit KDM2B to target genes to remove the activation mark H3K4me3. So far, we have not found an antibody against KDM2B that works in a ChIP assay with LCLs to test this point directly. Published ChIP studies of the KDM2B protein have mainly used tagged versions of KDM2B or antibodies which are not available commercially (49, 53, 65). It is important that at this stage we cannot rule out the possibility that EBNA3C interacts with and recruits other histone lysine demethylases, leading to the complete loss of H3K4me3.

The histone demethylase KDM2B is known to play important roles in tumorigenesis and self-renewal of cancer stem cells (66–68). Furthermore, KDM2B was demonstrated to contribute to the development of diffuse large B cell lymphoma (DLBCL) (69). In our study, we found that KDM2B plays a role in LCL survival, as the depletion of KDM2B decreased LCL cell viability (data not shown). Interestingly, KDM2B was also recently found to be silenced by EBV in Burkitt lymphoma cell lines, through DNA methylation (70). However, so far, no study has assessed the potential role of KDM2B in the development of EBV-positive Burkitt lymphoma. In our study, we found that EBNA3C HDmut is impaired in its interaction with KDM2B, suggesting a role of the TFGC motif in this interaction. However, it is important that we cannot rule out the possibility of KDM2B interacting elsewhere on EBNA3C or indirectly through additional factors.

Further studies will be needed to explore whether this mechanism of repression is conserved throughout the EBNA3 family of proteins and, more importantly, whether the same mechanism is used at other EBNA3C target genes. It is highly likely that different mechanisms of repression are involved, depending on the chromatin state of the target genes as well as on the presence of other EBV transcription factors. For instance, the EBNA3A protein has been shown to cooperate extensively with EBNA3C in the repression of thousands of cellular genes (40), and a two-step model has been proposed for the EBNA3A mode of action (56). Using the regulation of the *CXCL9* and *CXCL10* genes, Harth-Hertle and colleagues (56) described a model in which EBNA3A first interferes with EBNA2 recruitment, leading to a decrease of chromatin activation marks. Then, in a second step, the Polycomb proteins are recruited, leading to the deposition of H3K27me3 throughout the locus, suggesting that this epigenetic mark is a consequence rather than the driver of initial repression. However, we recently demonstrated that this is not always the case, as the recruitment of PRC2 and H3K27me3 are important for the initial repression of *STK39* by EBNA3A (71). Interestingly, whereas *STK39* is regulated only by EBNA3A (and does not involve EBNA3C), *CXCL9* and *CXCL10* are coregulated by both EBNA3A and EBNA3C, suggesting that different mechanisms of repression are used depending on the target genes and the cofactors involved.

EBNA3C is also involved in activation of transcription of some target genes. It has been found that EBNA3C binds to and activates the transcription of *AICDA* (72). Interestingly, investigation of the *AICDA* expression level during primary B cell infection with our different recombinant EBVs revealed that EBNA3C HDmut failed to fully activate its transcription (data not shown). Further work is needed to investigate and reveal the molecular mechanisms behind EBNA3C-mediated gene activation. It was, however, not surprising to find that EBNA3C HDmut also fails to activate gene transcription, as EBNA3C binding partners, such as HDAC1 and KDM2B, have been demonstrated to have transcriptional activation functions (73, 74).

Here we demonstrated that the two steps involved in EBNA3C-mediated repression of *COBLL1*, *ADAM28*, and *ADAMDEC1* are independent. Indeed, we showed that EBNA3C HDmut was incapable of completely removing H3K4me3 activation marks but was still fully able to recruit the Polycomb proteins to *COBLL1* and the *ADAM28-ADAMDEC1* locus and to induce the deposition of the repressive histone mark H3K27me3 at *COBLL1*. Furthermore, we showed that when the full removal of H3K27Ac at the *ADAM28-ADAMDEC1* locus was impaired by EBNA3C HDmut, the subsequent deposition of H3K27me3 was affected. This study provides evidence that removal of activation-associated chromatin marks is a prerequisite for efficient and full repression of EBNA3C-mediated gene targets.

In summary, EBNA3C interactions with some epigenetic modulators have been reported in the literature, but their biological relevance and their mechanisms are still poorly understood. Here we report that the EBV transcription factor EBNA3C interacts with the histone demethylase KDM2B, requiring the TFGC motif, previously known to be an RBPJ interaction site. Furthermore, we provide evidence that this interaction is important for rapid removal of the histone mark H3K4me3, leading to the repression of *COBLL1*, *ADAM28*, and *ADAMDEC1* gene expression. These findings further develop our understanding of these important EBV transcription factors, which reprogram cell gene transcription through epigenetic modifications and may drive the development of EBV-associated cancers.

## MATERIALS AND METHODS

### Cell culture.

Cells were cultured at 10% CO_2_ and 37°C in RPMI 1640 medium (Invitrogen) supplemented with 10% fetal calf serum (FCS), penicillin, and streptomycin. Puromycin was added at 1 μg/ml when selection was required. The activating ligand 4-hydroxytamoxifen (HT) was added to 400 nM where indicated.

### Recombinant EBV BACs.

WT and RBPJ binding mutant (BM) EBNA3C recombinant viruses were already constructed and used in a previous study (21). The creation of independent RBPJ interaction site EBNA3C mutant recombinant viruses was performed as previously described (21). Briefly, the N terminus of EBNA3C was cloned from the B95.8 EBV BAC (75) into modified pBlueScript II SK+ by XbaI digestion. Both motifs identified to be important for RBPJ interaction were mutated by In-Fusion PCR mutagenesis (Clontech). Each RBPJ interaction mutant fragment of EBNA3C was subcloned into the shuttle plasmid pKov-KanΔCm (76) and verified by DNA sequencing. EBV recombinants were created by RecA-based homologous recombination between the B95.8 EBV BAC and the shuttle plasmid, as previously described (76). EBNA3C Rev was constructed by homologous recombination between the RBPJ BM EBV BAC and a shuttle plasmid containing the EBNA3C WT sequence (77). RBPJ interaction mutant EBNA3C HDmut and W227S as well as EBNA3C Rev virus-producing 293 cell clones were established as previously described (77). Episome rescue of EBV BACs from virus-producing 293 cell lines was performed as previously described for low-molecular-weight DNA (78).

### Plasmids.

Open reading frames (ORFs) for EBNA3C WT, EBNA3C HDmut, EBNA3C W227S, and mCherry were cloned into a Gateway recombinational cloning system as previously described (79). Briefly, each ORF was cloned into pDONR207 (BP Clonase; Invitrogen) and verified by DNA sequencing. The ORFs were subsequently transferred (LR Clonase; Invitrogen) into pLenti CMV Puro DEST (Addgene). Small hairpin RNAs (shRNAs) targeting KDM2B or a nontarget (NT) control were cloned into the lentiviral vector pLKO.1. The different target sequences used were as follows: NT, 5′-CCTAAGGTTAAGTCGCCCTCG-3′; KDM2B-1, 5′-GCATGAGCTCTTGTACTTACA-3′; and KDM2B-2, 5′-CGGCCTTTACAAGAAGACATT-3′.

### Production of lentiviruses.

For viral packaging, lentivirus-based vectors pLKO.1 and pLenti CMV Puro DEST were cotransfected with helper plasmids psPAX2 and pMD2.G into 293T cells by the calcium phosphate precipitation method. Media containing viruses were collected 48 h after transfection.

### Viral infection of cells.

Primary B cells used in this study were isolated from buffy coat residues (UK Blood Transfusion Service) and infected as previously described (80). For lentiviral infections of established LCLs, cells were pelleted and resuspended with lentivirus in 293T medium with 8 μg/μl Polybrene. Cells were then centrifuged at 450 × *g* for 1.5 h at 22°C. The infected cells were then resuspended in RPMI medium and transferred to flasks.

### qPCR.

RNA was isolated from cells by use of an RNeasy minikit (Qiagen) with DNase digestion per the manufacturer's instructions. Reverse transcription of RNA into cDNA was performed using Superscript III first-strand synthesis supermix for qRT-PCR (Invitrogen). Ten nanograms of cDNA was run per qPCR, using a Platinum SYBR green qPCR supermix UDG kit (Invitrogen) and a QuantStudio 7 Flex real-time PCR machine. Primers used in the study were as follows: for *GNB2L1*, 5′-GCTTGCAGTTAGCCAGGTTC-3′ and 5′-GAGTGTGGCCTTCTCCTCTG-3′; for *ALAS1*, 5′-TCCACTGCAGCAGTACACTACCA-3′ and 5′-ACGGAAGCTGTGTGCCATCT-3′; for *COBLL1*, 5′-CTGTTCAGCTGACAACAGATCG-3′ and 5′-ACGTTGAACTCTCAGTGGTCCT-3′; for *ADAM28*, 5′-GTTGCAGGGACAATGGCACA-3′ and 5′-TGAGACGGCTGCAGGAACTG-3′; and for *ADAMDEC1*, 5′-CCTTGGTATGCCTGATGTTCCA-3′ and 5′-CAGCAGGCACTTTGGTTTCTGA-3′. The comparative threshold cycle (ΔΔ*C_T_*) method was used to calculate relative mRNA expression, with the housekeeping gene *GNB2L1* used as an endogenous control. Error bars in graphs show standard deviations for three triplicate qPCR replicates for each mRNA sample.

### Immunoprecipitation.

IPs were performed essentially as described previously (81). Briefly, 10^7^ LCL cells were harvested and lysed in immunoprecipitation (IP) buffer plus protease inhibitors (Roche Molecular Biochemicals). Cell extracts were then precleared with 55 μl of protein G-Sepharose beads (GE Healthcare) at 4°C for 1 h. Next, the protein of interest was immunoprecipitated from 200 μg of protein lysate overnight at 4°C. The antibodies used for IP were as follows: antibodies against RBPJ (rat monoclonal antibody 1F1; a gift from B. Kempkes, Helmholtz Zentrum München), BMI1 (A301-694A; Bethyl), SUZ12 (Ab12073; Abcam), and KDM2B (09-864; Millipore). Thirty microliters of protein G-Sepharose beads was added, incubated with rotation for 1 h at 4°C, and washed four times in IP buffer, and the immunopurified proteins were resolved by SDS-PAGE and detected by Western blotting. All IPs shown are representative examples of at least two independent experiments.

### Western immunoblotting.

Cells were washed twice in phosphate-buffered saline (PBS) and lysed in RIPA buffer for 30 min on ice. After centrifugation at 4°C for 10 min, the supernatant was removed, and the protein concentration was estimated calorimetrically using a Bio-Rad detergent-compatible assay. Protein samples (30 μg) were loaded onto SDS-polyacrylamide gels at a percentage appropriate for electrophoretic separation. Antibodies used for Western blotting were as follows: antibodies against EBNA3A (ab16126; Abcam), EBNA3B (clone 6C9; Allday lab), EBNA3C (clone A10; a gift from M. Rowe, University of Birmingham), EBNA1 (a gift from P. Farrell, Imperial College), EBNA2 (ab90543; Abcam), EBNA-LP (JF-186) (82), LMP1 (CS1-4; Dako), γ-tubulin (T6557; Sigma), RBPJ (J7A11-161; a gift from B. Kempkes, Helmholtz Zentrum München), KDM2B (09-864; Millipore), BMI1 (05-637; Millipore), SUZ12 (sc-46264; Santa Cruz), and mCherry (Ab183628; Abcam). In all blots, γ-tubulin was used as a loading control. The appropriate horseradish peroxidase (HRP)-conjugated antibodies were used as secondary antibodies (all from GE Healthcare). An ECL kit (Amersham Biosciences) was then used for visualization by autoradiography. In some cases, the membrane used for Western blotting was cut horizontally after protein transfer in order to facilitate multiple antibody probes and a single loading control for each blot used.

### Chromatin immunoprecipitation.

ChIP assay and qPCR analysis were performed as described previously (21). Primers used in these assays were as follows: for *MYOG*, 5′-GGAGAAAGAAGGGGAATCACA-3′ and 5′-GATAAATATAGCCAACGCCACA-3′; for *GAPDH*, 5′-CGCTCTCTGCTCCTCC-3′ and 5′-TTTCTCTCCGCCCGTCCAC-3′; for *ADAM* control, 5′-ACAGGAGCATGCACTCTTCA-3′ and 5′-GGCAATGTTCTGCTGCAA-3′; for the *ADAM* peak, 5′-CTTCATGGCTACAGACTCTTGG-3′ and 5′-CCTATGTCTCGCTTCCTGCT-3′; for *COBLL1* control, 5′-CCCTCCAGTATACCCCAGCT-3′ and 5′-ACCCCTTCTCTTTACTTGGCC-3′; for the *COBLL1* peak, 5′-CTGAGTAACAAGAGCGAAAGAG-3′ and 5′-ATCAGATGTGTTATGACTAACAGC-3′; and for the *COBLL1* TSS, 5′-GCCGCCGTCTCTACAAGGTCTA-3′ and 5′-CTACCCAGTAAACCCCACGG-3′. Antibodies used for ChIP experiments were as follows: antibodies against H3K27me3 (07-449; Millipore), H3K4me3 (17-614; Millipore), H3K27Ac (05-1334; Millipore), EBNA3C (ab16128; Abcam), RBPJ (ab25949; Abcam), BMI1 (A301-694A; Bethyl), and SUZ12 (Ab12073; Abcam). Input DNA was 5% of the DNA used for immunoprecipitations and was diluted to 2.5% prior to PCR quantification. Enrichment relative to the input level was calculated using four 5-fold-dilution series, and error bars were calculated as standard deviations for triplicate PCRs for both input and IP samples. All ChIPs shown are representative of at least two independent experiments, each performed on LCLs established by two independent primary B cell infections.

### Flow cytometry.

Cell proliferation assays were performed as described previously (12), by measuring the incorporation of the nucleotide analogue EdU into DNA during a 2-h pulse and the DNA content by use of FxCycle Far Red DNA stain. Cell fluorescence was measured on an LSR II flow cytometer (Becton Dickinson).

### Ethics statement.

The buffy coat residues used in this study for the isolation of CD19^+^ primary B cells were purchased from the UK Blood Transfusion Service. As these were derived from anonymous volunteer blood donors, no ethical approval is required.
